# Association between filaggrin gene mutations and the clinical features of molluscum contagiosum: The Yamanashi Adjunct Study of the Japan Environment and Children's Study

**DOI:** 10.1111/1346-8138.17157

**Published:** 2024-02-27

**Authors:** Reiji Kojima, Kunio Miyake, Ryoji Shinohara, Megumi Kushima, Hideki Yui, Sanae Otawa, Sayaka Horiuchi, Hiroshi Yokomichi, Yuka Akiyama, Tadao Ooka, Zentaro Yamagata, the Yamanashi Adjunct Study of the Japan Environment and Children's Study Group, Anna Kobayashi, Takeshi Inukai, Kyoichiro Tsuchiya, Hirotaka Haro, Masanori Wako, Takahiko Mitsui, Kenji Kashiwagi, Daijyu Sakurai, Koichiro Ueki, Sumire Ono

**Affiliations:** ^1^ Department of Health Sciences, School of Medicine University of Yamanashi Chuo Yamanashi Japan; ^2^ Department of Epidemiology and Environmental Medicine, School of Medicine University of Yamanashi Chuo Yamanashi Japan; ^3^ Center for Birth Cohort Studies University of Yamanashi Chuo Yamanashi Japan

**Keywords:** atopic dermatitis, filaggrin gene mutations, molluscum contagiosum, time to resolution, treatment modalities

## Abstract

Previous studies have reported swimming, atopic dermatitis, and filaggrin (FLG) gene mutations as risk factors for molluscum contagiosum (MC) infection. FLG gene mutations impair skin barrier function. The aim of this study was to determine the impact of FLG mutations on the incidence and clinical features of MC. We used data from 2036 children who participated in the Yamanashi Adjunct Study of the Japan Environment and Children's Study, a prospective, birth cohort study. A questionnaire for caregivers (when children were 4 and 8 years of age) asked about clinical features including previous MC incidence and treatment, number of MC lesions at first visit, and time to resolution. Participants underwent genotyping to detect six FLG mutations that are common in the Japanese population. A logistic regression model was used to analyze the association between MC incidence and FLG mutations, adjusted for potential confounders. The cumulative incidence of MC at age 8 years was 47.1%. Among participants with a history of MC, 67.6% had undergone curettage. FLG mutation was a significant risk factor for MC incidence (adjusted odds ratio [aOR] 1.69, 95% confidence interval [CI] 1.18–2.42). Swimming and atopic dermatitis were also significant risk factors for MC. There was no significant association between FLG mutation and the number of MC lesions at the first visit or the time to resolution of lesions. FLG mutation is a risk factor for MC incidence; however, FLG mutations do not affect the number of MC lesions at presentation or the time to resolution.

## 1 INTRODUCTION

Molluscum contagiosum (MC) is a common viral skin infection in children, which is characterized by dome‐shaped umbilicated papules, typically measuring 1–5 mm in diameter.^1^, ^2^ MC, which is caused by the molluscum contagiosum virus, is benign and resolves spontaneously in immunocompetent individuals. MC is usually transmitted by direct contact, fomites, and autoinoculation. The natural history of MC begins with the onset of a few MC papules, followed by an increase in the number of papules and development of surrounding eczema‐like lesions (molluscum dermatitis).^2^ After several months, the spontaneous regression phase begins, which involves erythema and swelling of the MC lesions, referred to as the “beginning of the end” sign.^2^, ^3^ Lesion duration varies from a few months to several years.^4^ During this time, treatment may be required for worsening eczema and bacterial superinfections or for aesthetic reasons.^2^ Several MC treatment modalities can be used, including physical methods (e.g., curettage and cryotherapy), chemical methods (e.g., cantharidin and salicylic acid), and immunomodulatory methods (e.g., imiquimod).^2^ However, no consensus has been established regarding the standard of care.^2^, ^5^ Therefore, treatment methods are currently determined by the patient's clinical status and can vary according to the region and the physician's specialty.^6^, ^7^

Epidemiological studies of MC are limited. A systematic review by Olsen et al.^8^ found a prevalence of 5%–11%, with children aged 1–4 years most commonly affected. However, the prevalence may be underestimated because many studies have focused on MC‐affected children who visited a medical clinic, and, therefore, have failed to include MC patients who did not seek medical treatment.^8^ Risk factors for MC include swimming,^9^, ^10^ affected siblings,^4^, ^11^ atopic dermatitis (AD),^3^, ^11^, ^12^, ^13^, ^14^, ^15^, ^16^, ^17^ and filaggrin (FLG) gene mutations.^18^ Swimming and siblings living together can facilitate transmission of the virus by direct contact with infected persons and fomites. AD is manifested by immunological changes in the skin and impaired skin barrier function^9^, ^10^ and several reports show a significant association between AD and MC. FLG mutations are also responsible for impaired skin barrier function.^19^ However, very few studies have investigated the association between FLG mutations and MC. Although Manti et al.^18^ found that FLG mutations were significantly associated with AD‐associated MC, their study did not adjust for risk factors such as swimming or investigate the impact of FLG mutations on the clinical course of the disease.

The aim of this study was, thus, to determine the population‐based cumulative incidence of MC and to assess the impact of FLG mutations on the incidence and clinical features of MC.

## 2 METHODS

### 2.1 Study design and participants

This study was part of the Yamanashi Adjunct Study of the Japan Environment and Children's Study (JECS). The protocol and baseline data of the JECS have been reported elsewhere.^20^, ^21^ Briefly, the JECS is an ongoing, nationwide, birth cohort study that includes more than 100 000 pregnant women who were recruited between January 2011 and March 2014 and enrolled at 15 JECS Study Areas covering a wide geographic area in Japan. For the present study, 2099 participants who presented at the 8‐year follow‐up of the JECS in the Yamanashi Prefecture were approached for the Yamanashi Adjunct Study of JECS, including genotyping. Questionnaires for caregivers (when children were 4 and 8 years of age) were used to ask about demographic characteristics (sex, siblings) and MC features at 8 years of age (history of MC, diagnosing physician, number of MC lesions at first visit, treatment, reasons for curettage, swimming, and history of AD). The JECS protocol was reviewed and approved by the Ministry of the Environment's Institutional Review Board on Epidemiological Studies (IRB number: 100910001). The study was approved by the Institutional Review Board of Yamanashi University (No. 2070, 2218). Written informed consent was obtained from all participants. This study was based on the JECS datasets jecs‐ta‐20 190 930 (2022.11.29ver) and jecs‐qa‐20 210 401(2023.3.16ver), which were released in October 2019 and April 2021, respectively.

### 2.2 Variables

#### 2.2.1 Outcomes

The primary outcome of this study was the cumulative incidence of MC in children at 8 years of age. The questionnaire asked whether participants had ever had MC, with a “yes” response defined as incident MC. The number of MC lesions at the first clinic visit and time to resolution were used as subsidiary outcomes. Time to resolution was defined as the time between the first clinic visit and resolution. Response options for the question on modalities of treatment for MC included curettage, topical treatment (salicylic acid, povidone iodine), internal treatment (coix seed extract powder, *Yokuinin*, [a Chinese herbal medicine]), other, and no treatment.

#### 2.2.2 Genotyping

We screened for R501X (rs145828067), Q1701X (rs145738429), S2554X (rs1312278747), S2889X (rs540453626), S3296X (rs761212672), and K4022X (rs146466242), which are highly frequent missense mutations in the FLG gene in the Japanese population.^22^, ^23^, ^24^ The presence of at least one mutation was defined as an FLG mutation. Genomic DNA was extracted from whole blood using the FlexiGene DNA kit (Qiagen). FLG genotypes were determined with the Biomark HD system (Standard BioTools), according to the manufacturer's protocol.

### 2.3 Statistical analyses

Participants with a missing history of MC (*n* = 4) were excluded and the cumulative incidence of MC at 8 years of age was calculated (*n* = 2032). To examine the association between MC incidence and FLG mutations, a logistic regression model was used. Swimming, affected siblings, and AD were included as adjustment variables in the logistic model. For the analysis, 1757 participants were included, excluding those with missing history of MC (*n* = 4) and no FLG mutation data (*n* = 275). AD was defined based on a partially modified version of the International Study of Asthma and Allergies in Childhood questionnaire for 6–7‐year‐olds.^25^, ^26^, ^27^ Moreover, subgroup analyses were performed to examine modification of the effect of swimming attendance on the incidence of MC by AD or FLG mutations. We also tested whether the association between swimming attendance and the incidence of MC is changed by AD or FLG mutations using the Wald test. We used mediation analysis^28^ to determine the proportion of the association between FLG mutations and the incidence of MC, which is potentially mediated by AD. Using PROC CAUSALMED in SAS (SAS Inc.), we estimated the natural direct effects and natural indirect effects of mediators after controlling for all covariates used in the adjusted model. The association between FLG mutations and the number of MC lesions and time to resolution was examined using the Kruskal‐Wallis test with the response items as ordinal variables. Statistical analysis was performed using SAS version 9.4 (SAS Institute). A *p*‐value of <0.05 was considered statistically significant.

## 3 RESULTS

Study participant characteristics are shown in Table 1. Of the participants, 50.9% were female. At 4 years of age, 22.5% swam at least once a month, and at 8 years of age, 32.8% were attending swimming classes. At 4 years of age, 17.5% of participants had AD symptoms. The cumulative incidence of MC at 8 years of age was 47.1%, and 8.3% of patients had FLG mutations (details are shown in Table S1).

**TABLE 1:** Characteristics of study participants (*n* = 2032).

	Number	(%)
Sex		
Male	997	49.1
Female	1035	50.9
Swimming at 8 years		
Yes	661	32.8
No	1357	67.2
Swimming frequency at 4 years		
None	408	21.2
Only a few times in summer	1084	56.2
Once a month	49	2.5
Two to three times a month	99	5.1
Once a week	153	7.9
More than twice a week	135	7.0
Younger siblings		
Yes	611	31.6
No	1321	68.4
Older siblings		
Yes	921	47.7
No	1011	52.3
Atopic dermatitis symptoms at age 4 years		
Yes	338	17.5
No	1598	82.5
Eczema in favored areas^a^		
Yes	278	14.4
No	1658	85.6
Nocturnal sleep disturbance due to eczema		
Yes	96	5.0
No	1840	95.0

a Intertriginousareas such as the flexed side of the elbow and knee joints.

Table 2 shows the odds ratios (ORs) for risk factors of MC incidence. FLG mutation was a significant risk factor for MC incidence (crude OR, 1.66; 95% confidence interval [CI] 1.17–2.34). This association remained after adjustment for swimming, siblings, and AD (adjusted OR [aOR], 1.69; 95% CI 1.18–2.42). Swimming at 8 years of age was a significant risk factor for MC (aOR, 1.42; 95% CI 1.15–1.75). More frequent swimming at 4 years of age was a risk factor for MC incidence, with more frequent swimming associated with a higher risk (*p* < 0.01 for trend). AD was also a risk factor for MC incidence (aOR, 1.43; 95% CI 1.10–1.85). In contrast, siblings were not significantly associated with MC incidence. Details on FLG mutations and MC incidence are presented in Table S2. Subgroup analyses stratified by AD or FLG mutations showed that there was an increased risk of swimming attendance in participants with AD or FLG mutations compared with those without but there was no significant interaction (Table S3). The mediation analysis showed that AD did not significantly mediate the relationship between FLG mutations and the incidence of MC (4.6%, *p* = 0.42; Table S4).

**TABLE 2:** Odds ratios for factors related to molluscum contagiosum.

	Crude OR	95% CI	Adjusted OR	95% CI
Swimming attendance				
No	ref		ref	
Yes	**1.34**	**1.09, 1.63**	**1.42**	**1.15, 1.75**
Younger siblings	1.11	0.90, 1.37	1.07	0.86, 1.34
Older siblings	0.92	0.76, 1.12	0.95	0.77, 1.17
Atopic dermatitis	**1.39**	**1.08, 1.78**	**1.43**	**1.10, 1.85**
FLG mutation	**1.66**	**1.17, 2.34**	**1.69**	**1.18, 2.42**
Swimming frequency				
None	ref		ref	
Only a few times in summer	1.14	0.89, 1.46	1.15	0.90, 1.48
Once a month	1.65	0.88, 3.07	1.86	0.98, 3.56
Two to three times a month	1.41	0.88, 2.24	1.38	0.86, 2.21
Once a week	**1.65**	**1.09, 2.48**	**1.60**	**1.06, 2.43**
More than twice a week	**1.73**	**1.14, 2.63**	**1.66**	**1.09, 2.54**
Younger siblings	1.11	0.90, 1.37	1.09	0.87, 1.37
Older siblings	0.92	0.76, 1.12	0.92	0.75, 1.14
Atopic dermatitis	**1.39**	**1.08, 1.78**	**1.38**	**1.07, 1.79**
FLG mutation	**1.66**	**1.17, 2.34**	**1.67**	**1.16, 2.40**

*Note*: Boldface indicates statistical significance (*p* < 0.05).

Abbreviations: CI, confidence interval; FLG, filaggrin; OR, odds ratio.

Table 3 shows the clinical features of participants with a history of MC. Of these participants, 64.4% had fewer than 10 MC lesions at the first visit and 32% had 10–30 MC lesions at the first visit. In total, more than 95% of these patients had fewer than 30 MC lesions at first visit. Dermatologists and pediatricians diagnosed MC in 71.9% and 10.4% of participants respectively, while 14.5% of participants were not diagnosed by a physician. Approximately 70% of participants were treated by curettage: 57.5% of participants underwent curettage as the first treatment and 10.1% of participants underwent curettage after the second visit. The most common time to resolution was less than 2 weeks (in approximately 30% of cases). The first treatment parsed by physician specialty is shown in Table S5. There was a significant difference between dermatologists (62.4%) and pediatricians (26.4%) for curettage, and between dermatologists (13.6%) and pediatricians (34.9%) for “no treatment” (*p* < 0.01).

**TABLE 3:** Clinical characteristics of study participants with molluscum contagiosum.

	Number	(%)
Number of MC lesions at the first visit		
<10	607	64.4
10 ≤ 30	302	32.0
30 ≤ 60	30	3.2
60 ≤	4	0.4
Diagnosing physician		
Dermatologist	786	71.9
Pediatrician	114	10.4
None	158	14.5
Not remember	9	0.8
Both dermatologist and pediatrician	26	2.4
Treatment at the first visit		
Curettage	512	57.5
Other than curettage^a^	230	25.8
No treatment	149	16.7
Treatment including subsequent treatments		
Curettage at the first visit	512	57.5
Other than curettage	178	20.0
No treatment	111	12.5
Curettage after the second visit	90	10.1
Time to resolution		
<2 week	268	29.0
2 weeks ≤ 1 month	202	21.9
1 month ≤ 2 months	116	12.6
2 months ≤ 6 months	144	15.6
6 months ≤ 1 year	102	11.1
1 year ≤	52	5.6
Not cured	39	4.2

Abbreviation: MC, molluscum contagiosum.

^a^ Including cryotherapy (*n* = 24).

**FIGURE 1:**
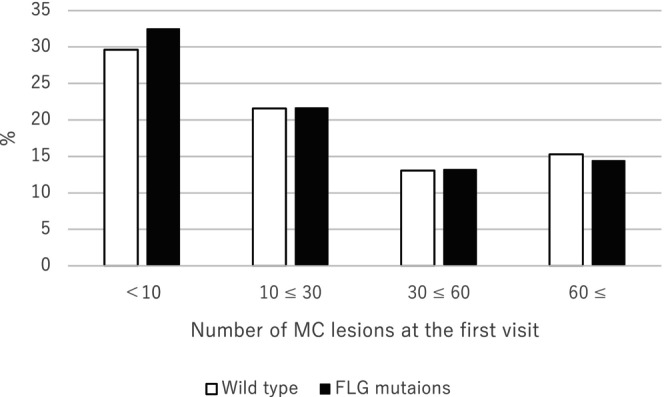
Relationship between the number of molluscum contagiosum (MC) lesions at the first visit and filaggrin (FLG) mutations. Data were analyzed by Kruskal–Wallis test, *p* = 0.74.

**FIGURE 2:**
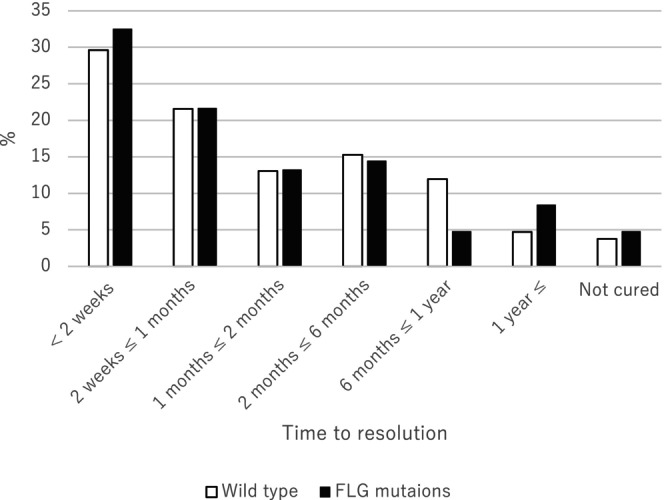
Relationship between time to resolution and filaggrin (FLG) mutations. Data were analyzed by Kruskal–Wallis test, *p* = 0.62.

Figure 1 shows the relationship between the number of MC lesions at the first visit and FLG mutations. No significant differences in the number of MC lesions at the first visit were found between participants with FLG mutations and those without. Figure 2 shows the relationship between the time to resolution of MC and FLG mutations. There was no significant difference in time to resolution of MC between participants with and without FLG mutations. There was also no significant difference between the number of MC lesions at first visit or time to resolution of MC in participants with AD and those without (Figures S1 and S2).

**FIGURE 3:**
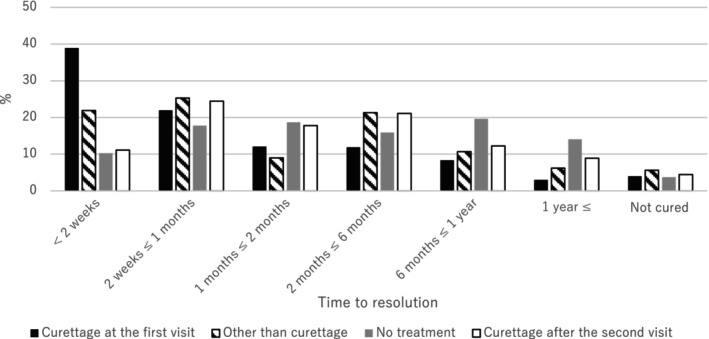
Time to resolution for each treatment modality. Data were analyzed by Kruskal–Wallis test, *p* < 0.01.

Figure 3 shows the time to resolution of MC for each treatment modality. There were significant differences between treatment modalities and time to resolution of MC (*p* < 0.01). Participants who had a curettage at the first visit had the highest proportion of resolution within 2 weeks. For treatments other than curettage, the most common time to resolution was 2 weeks to 1 month. For “no treatment”, the most common time to resolution was 6 months to 1 year. Figure S3 shows the reasons for choosing curettage among participants who underwent the procedure. “Physician's recommendation” was the most common reason for both the first and second or subsequent curettage (84.1% and 60.0% respectively). “Due to increased MC lesions” and “not cured by the first treatment” were more common for curettage at the second or subsequent visit, compared with curettage at the first visit. “Nurseries/schools encouraged to remove” was a reason given by 8% of those who underwent curettage as a first treatment. More than 20% of those who underwent curettage both at the first visit and at the second or subsequent visit gave a reason for curettage as “Told MC needs to be removed to get into the pool” (21.0% and 25.6% respectively). Figure S4 shows the treatment desired by the caregivers of participants who underwent treatment. Curettage was selected by 21.7%, followed by salicylic acid (13.7%), and no treatment (6.7%). “No specific preference” was the most common response (36.6%).

## 4 DISCUSSION

In our population‐based study, the cumulative incidence of MC at 8 years of age was 47.1%. FLG mutation was a risk for the incidence of MC, but there was no significant association between FLG mutation and the number of MC lesions at the first visit or the time to resolution.

The cumulative MC incidence in our study is higher than the previously reported MC prevalence, but this discrepancy may be due to differences in epidemiological indices and study methodology, in addition to geographical variation. Our study examined the cumulative incidence of MC reported by caregivers in children aged 8 years, rather than the prevalence. Koning et al.^29^ reported that the cumulative incidence of MC up to 14 years of age in the Netherlands was 17%. Hayashida et al.^13^ reported that, in Japan, the cumulative incidence of physician‐diagnosed MC was 31.1% at 4–6 years of age. An Australian seroepidemiology study also found that the prevalence of antibodies to MC was 31.1% at 4–6 years of age.^30^ Epidemiological studies of MC may underestimate prevalence because the disease is self‐limiting, and most studies were hospital‐based.^8^

The prevalence of FLG mutations in our study was 8%, which is comparable to previous reports on the general Japanese population.^22^ In our study, FLG mutation was a significant risk for MC incidence, which was consistent with the previous report by Manti et al.^18^ In contrast to the Manti et al. study, we adjusted our analysis for other known MC risk factors and the association remained significant. The MC virus infects the epidermis and replicates in the cytoplasm of cells. Because FLG is responsible for skin barrier function, mutations in FLG may lead to impaired skin barrier function^19^ and thus be a risk factor for MC. In our study, swimming and AD were also risk factors for MC, consistent with previous reports.^3^, ^9^, ^10^, ^11^, ^12^, ^13^, ^14^, ^15^, ^16^, ^17^ The mediation analysis showed no significant mediation by AD in the relationship between FLG mutations and the incidence of MC. AD and FLG mutations do not always overlap. Our results suggest that the mechanism for the development of MC due to FLG mutations may be different from the development of MC due to AD, therefore, further research on this issue is warranted. Our study also showed for the first time that swimming is associated with an increased MC risk with increasing swimming frequency in a community population rather than in a patient population. A stratified analysis showed no significant interaction between AD or FLG mutations and swimming attendance. Regardless of the condition with or without AD and FLG mutations, we suggest direct skin‐to‐skin contact and the sharing of towels and other objects should be avoided when swimming.^31^

In our study, FLG mutation was not significantly associated with the number of MC lesions at the first visit. The number of MC lesions at the first visit may depend on whether caregivers visited a healthcare provider, which might be influenced by differences in the healthcare system such as accessibility of healthcare. There is conflicting data on the association between AD and the number of MC lesions at first diagnosis. One previous study reported significantly higher numbers of MC lesions at diagnosis in children with both MC and AD,^3^ while other studies reported no significant association.^11^, ^17^

In our study, there was no significant association between FLG mutation and time to resolution of lesions. However, our study was observational, and more than half of the children were treated with curettage. Thus, the effect of FLG mutations on time to resolution might have been masked by treatment. More studies are needed to assess the effect of FLG mutations on the natural history of MC.

We found that treatment modality had a significant effect on the time to resolution of MC. Although curettage resulted in the shortest time to resolution, there is a possibility of reverse causation because this was an observational study. Treatment other than curettage may have been chosen if the number of MC lesions was high. A US study reported no significant difference in time to resolution according to treatment modalities. However, in that study, no treatment for MC was chosen in 73% of cases, and curettage was chosen in only 4% of patients.^32^ Therefore, those data cannot be compared with the results of our study. There may be both medical and social reasons why curettage is the major treatment modality for MC in Japan. In our study, dermatologists were more likely than pediatricians to choose excision, while pediatricians were more likely than dermatologists to choose no treatment. This result is consistent with data from the US showing differences in treatment choice for MC, based on physician specialty.^7^ In Japan, caregivers are free to choose a clinic to attend, rather than their general practitioner, and 72% of participants in this study were diagnosed with MC by a dermatologist. Furthermore, the most common reason for curettage was “physician recommendation” (84%), and the most common response for the treatment the caregiver desired was “no specific preference” (37%), followed by curettage (22%), and no treatment (7%). These data suggest that the decision to treat MC in Japan is largely influenced by the preference of the physician. In addition, there could be social demands that encourage the choice of curettage. Among participants who underwent curettage, the reasons for curettage were “Nurseries/schools encouraged to remove” (8%) and “Told MC needs to be removed to get into the pool” (21%). However, the Japanese Society of Pediatric Dermatology and other societies have stated that “MC is known to be transmitted through direct skin‐to‐skin contact or through swimming board/kickboard. However, the virus is not known to be transmitted through water, therefore, it is not a problem for infected patients to get into the pool.”^31^

The strength of this study is that it determined the cumulative incidence of MC in a population‐based setting. Hospital‐based studies have not been able to detect MC‐affected children who do not see a doctor, whereas this study was able to detect those children (approximately 15%). Our study also analyzed the effect of FLG mutations on MC incidence, adjusting for risk factors, including swimming.

Our study had several limitations. First, MC incidence was caregiver‐reported. However, the likelihood of misclassification regarding MC incidence is low because of the consistency of responses regarding treatment of MC. The time to resolution of MC was also reported by caregivers, which could lead to a recall bias. However, caregivers were not informed whether participants had the FLG mutation, which suppresses bias. As the current study was observational, most participants underwent treatment. Therefore, we could not assess the impact of FLG mutations on the natural history of MC severity. Studies in populations without medical intervention are needed to determine the impact of FLG mutations on the natural history of MC.

In conclusion, the cumulative incidence of MC at 8 years of age in this population‐based study was 47.1%. FLG mutation was a risk factor for MC incidence. In regions where curettage was the major treatment for MC, there was no significant difference in the number of MC lesions and time to resolution in patients with or without FLG mutations.

## CONFLICT OF INTEREST STATEMENT

None declared.

## Supporting information


Appendix S1.

Figure S1.

Figure S2.

Figure S3.

Figure S4.
