# Supplementary material for: Association between filaggrin gene mutations and the clinical features of molluscum contagiosum: The Yamanashi Adjunct Study of the Japan Environment and Children's Study
Source: J Dermatol. 2024 Feb 27;51(4):484–90. doi: 10.1111/1346-8138.17157 (PMC11484127; doi:10.1111/1346-8138.17157)
Supplement: Supplementary file 1 — Appendix S1. [file JDE-51--s003.docx]

Figure captions

Figure S1. Relationship between the number of MC lesions at the first visit and atopic dermatitis.

Data were analyzed by Kruskal-Wallis test, p=0.06. AD, atopic dermatitis; MC, molluscum contagiosum.

Figure S2. Relationship between time to resolution and atopic dermatitis.

Data were analyzed by Kruskal-Wallis test, p=0.91. AD, atopic dermatitis.

Figure S3. Reasons for choosing curettage.

MC, molluscum contagiosum.

Figure S4. Treatment desired by caregivers of participants who underwent treatment.

| Table S1. Number of children with FLG mutation (n = 1757) | | | | | |  |  |
| --- | --- | --- | --- | --- | --- | --- | --- |
| FLG mutation | R501X | Q1701X | S2554X | S2889X | S3296X | K4022X ^b^ | Compound ^a^ |
| n | 1 | 96 | 93 | 1 | 11 | 39 | 145 |
| (%) | 0.06 | 5.46 | 5.29 | 0.06 | 0.63 | 2.22 | 8.25 |
| FLG: filaggrin. ^a^ This includes (S2554X/Q1701X, n= 92) and (S2554X/Q1701X/R501X/K4022X/S3296X, n=1).  ^b^ This includes homozygote (n=1), otherwise, heterozygote. | | | | | | | |

| Table S2. FLG mutation and MC infection (n=1757) | | | | |  |  |  |  |  |
| --- | --- | --- | --- | --- | --- | --- | --- | --- | --- |
|  |  |  | MC (+) | |  | MC (-) | |  |  |
|  |  |  | n | (%) |  | n | (%) |  | *P* value ^b^ |
| R501X |  | |  |  |  |  |  |  |  |
|  | Wild-type FLG | | 827 | 47.1 |  | 929 | 52.9 |  | 0.471 |
|  | FLG mutation | | 1 | 100 |  | 0 | 0 |  |  |
| Q1701X |  | |  |  |  |  |  |  |  |
|  | Wild-type FLG | | 776 | 46.72 |  | 885 | 53.28 |  | 0.155 |
|  | FLG mutation | | 52 | 54.17 |  | 44 | 45.83 |  |  |
| S2554X |  | |  |  |  |  |  |  |  |
|  | Wild-type FLG | | 778 | 46.75 |  | 886 | 53.25 |  | 0.188 |
|  | FLG mutation | | 50 | 53.76 |  | 43 | 46.24 |  |  |
| S2889X |  | |  |  |  |  |  |  |  |
|  | Wild-type FLG | | 828 | 47.15 |  | 928 | 52.85 |  | 1 |
|  | FLG mutation | | 0 | 0 |  | 1 | 100 |  |  |
| S3296X |  | |  |  |  |  |  |  |  |
|  | Wild-type FLG | | 822 | 47.08 |  | 924 | 52.92 |  | 0.62 |
|  | FLG mutation | | 6 | 54.55 |  | 5 | 45.45 |  |  |
| K4022X |  | |  |  |  |  |  |  |  |
|  | Wild-type FLG | | 799 | 46.51 |  | 919 | 53.49 |  | 0.0023 |
|  | FLG mutation | | 29 | 74.3 |  | 10 | 25.6 |  |  |
| Compound ^a^ | |  |  |  |  |  |  |  |  |
|  | Wild-type FLG | | 743 | 46.09 |  | 869 | 53.91 |  | 0.0038 |
|  | FLG mutation | | 85 | 58.62 |  | 60 | 41.38 |  |  |

FLG: filaggrin; MC, molluscum contagiosum. ^a^ This includes (S2554X/Q1701X, n= 92) and

(S2554X/Q1701X/R501X/K4022X/S3296X, n=1). ^b^ Data were analyzed by chi-square test or Fisher's exact test.

| Table S3. Adjusted odds ratios of MC incidence in relation to swimming attendance by atopic dermatitis or FLG mutations | | | | | |  |
| --- | --- | --- | --- | --- | --- | --- |
|  |  | Swimming attendance | |  |  |  |
|  |  | Adjusted OR ^a^ | 95％CI | | P for interaction ^b^ |  |
| Atopic dermatitis | |  |  |  |  |  |
|  | No | **1.37** | **1.09,** | **1.72** | **0.44** |  |
|  | Yes | **1.75** | **1.01,** | **3.04** |  |  |
|  |  |  |  |  |  |  |
| FLG mutations | |  |  |  |  |  |
|  | No | **1.38** | **1.11,** | **1.71** | **0.40** |  |
|  | Yes | 2.07 | 0.93, | 4.60 |  |  |
| OR: odds ratio; CI: confidence interval. Boldface indicates statistical significance (p < 0.05). ^a^ Adjusted model includes younger siblings and older siblings. ^b^ Swimming attendance and atopic dermatitis or FLG mutations. | | | | | |  |
|  |  |  |  |  |  |  |
|  |  |  |  |  |  |  |
|  |  |  |  |  |  |  |

| Table S4. Estimates of direct and indirect effects (mediated through atopic dermatitis) of the association between FLG mutations and MC incidence | | | | | |
| --- | --- | --- | --- | --- | --- |
|  |  |  |  |  |  |
|  | Estimated value | p value |  | Proportion mediated (%) | p value |
| Total effect | 0.09 | 0.0058 |  |  |  |
| Natural indirect effects | 0.0042 | 0.42 |  | 4.6 | 0.42 |
| Natural direct effects | 0.086 | 0.008 |  |  |  |
| Models: Adjusted for younger siblings and older siblings. | | |  |  |  |

| Table S5. Physician specialty and treatment modalities | | | | | | | |  |
| --- | --- | --- | --- | --- | --- | --- | --- | --- |
|  | Curettage | |  | Other than curettage | |  | No treatment | |
| Physician specialty | Number | (%) |  | Number | (%) |  | Number | (%) |
| Dermatologist | 468 | 62.4 |  | 180 | 24.0 |  | 102 | 13.6 |
| Pediatrician | 28 | 26.4 |  | 41 | 38.7 |  | 37 | 34.9 |

Data were analyzed by chi-square test, p<0.01.
