# Supplementary figures and images for: Association between filaggrin gene mutations and the clinical features of molluscum contagiosum: The Yamanashi Adjunct Study of the Japan Environment and Children's Study
Source: J Dermatol. 2024 Feb 27;51(4):484–90. doi: 10.1111/1346-8138.17157 (PMC11484127; doi:10.1111/1346-8138.17157)

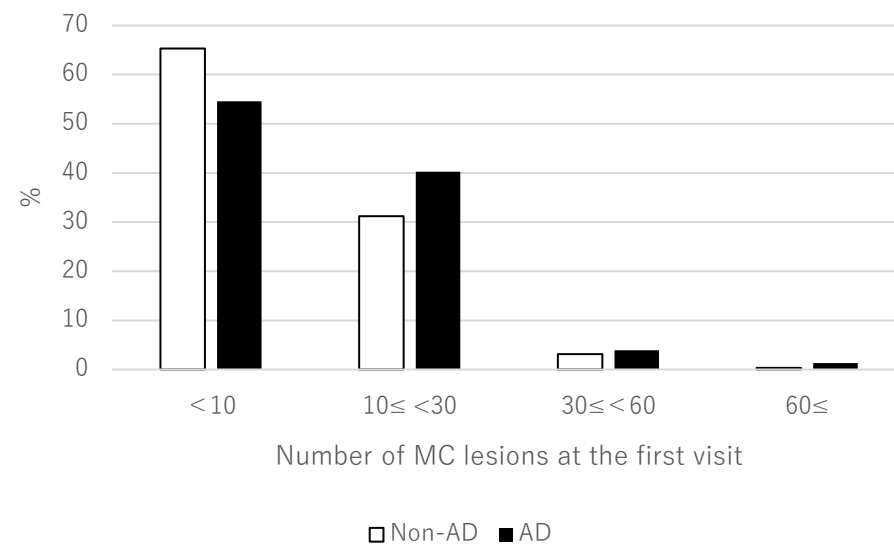

Figure S1

Supplement: Supplementary file 2 — Figure S1. [file JDE-51--s004.pdf]

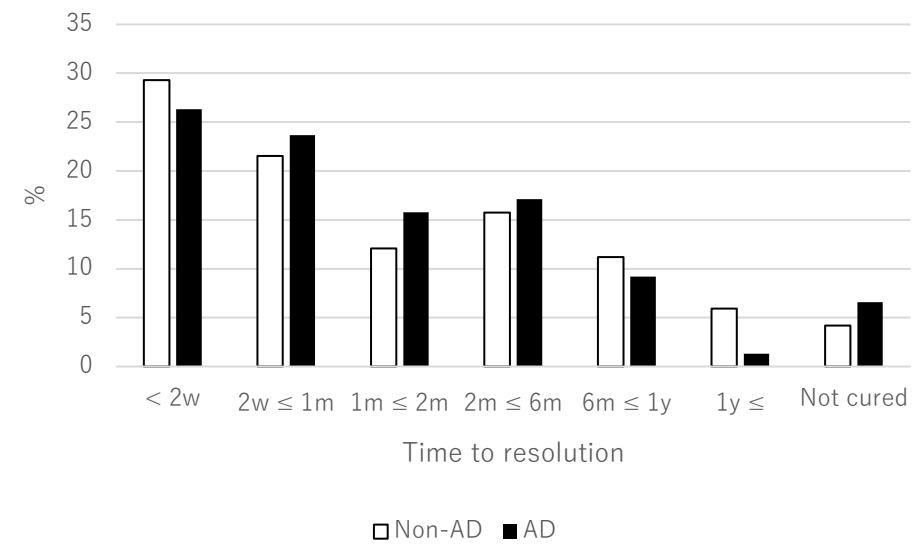

Figure S2

Supplement: Supplementary file 3 — Figure S2. [file JDE-51--s005.pdf]

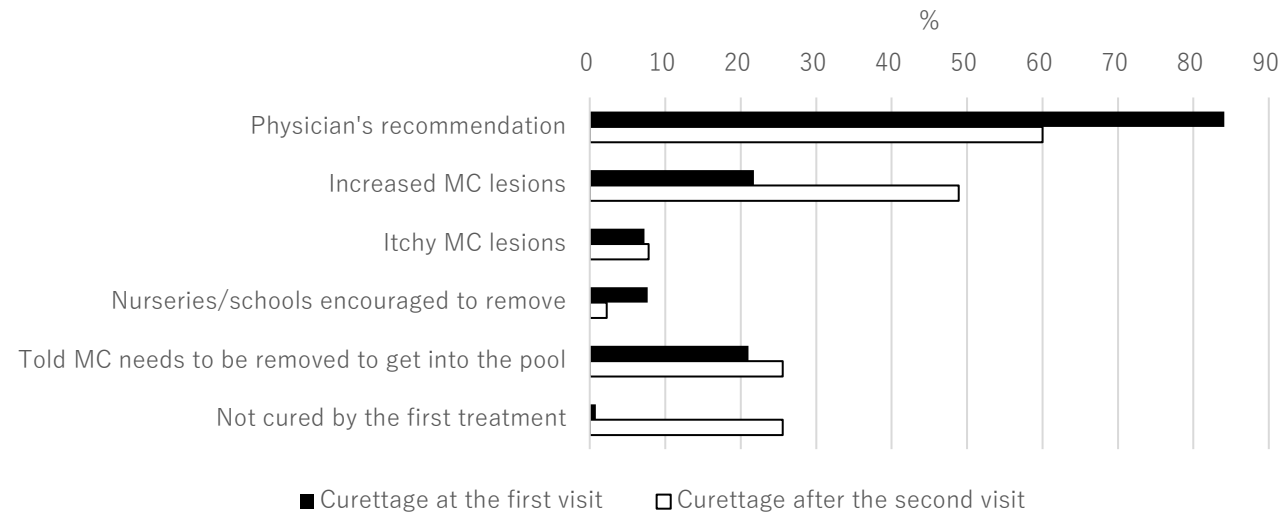

Figure S3

Supplement: Supplementary file 4 — Figure S3. [file JDE-51--s002.pdf]

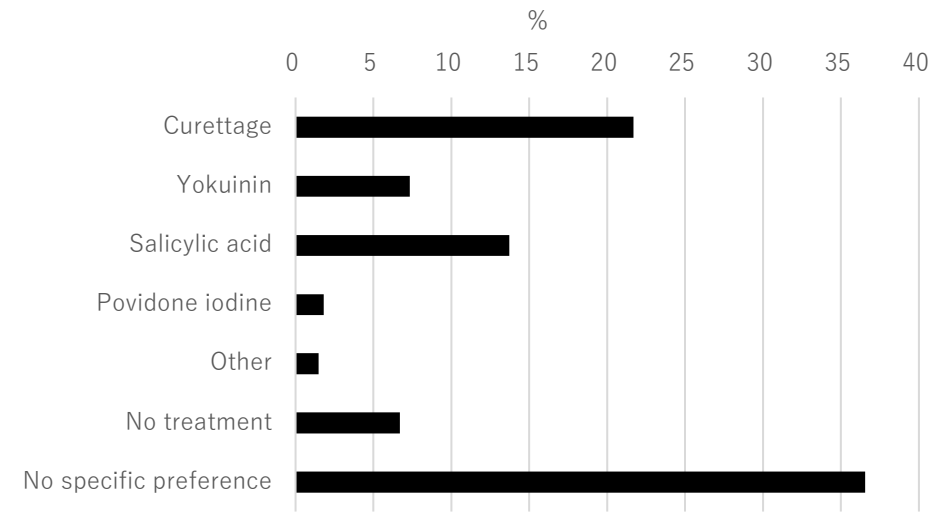

Figure S4

Supplement: Supplementary file 5 — Figure S4. [file JDE-51--s001.pdf]
